# The Paleoecology, Habitats, and Stratigraphic Range of the Enigmatic Cretaceous Brachiopod *Peregrinella*

**DOI:** 10.1371/journal.pone.0109260

**Published:** 2014-10-08

**Authors:** Steffen Kiel, Johannes Glodny, Daniel Birgel, Luc G. Bulot, Kathleen A. Campbell, Christian Gaillard, Roberto Graziano, Andrzej Kaim, Iuliana Lazăr, Michael R. Sandy, Jörn Peckmann

**Affiliations:** 1 Georg-August-Universität Göttingen, Geowissenschaftliches Zentrum, Abteilung Geobiologie, Göttingen, Germany; 2 Deutsches GeoForschungsZentrum GFZ, Sektion 4.2, Anorganische und Isotopengeochemie, Telegrafenberg, Potsdam, Germany; 3 Universität Wien, Erdwissenschaftliches Zentrum, Department für Geodynamik und Sedimentologie, Wien, Austria; 4 FRE CNRS 2761, Centre de Sédimentologie-Paléontologie, Université de Provence, Marseille, France; 5 University of Auckland, Geology Programme, School of Environment Science, Auckland, New Zealand; 6 Université de Lyon-1, UMR CNRS 5125 Paléoenvironnements et Paléobiosphère, Villeurbanne, France; 7 Dipartimento di Scienze della Terra, dell'Ambiente e delle Risorse, Università di Napoli Federico II, Largo S. Marcellino, Napoli, Italia; 8 Instytut Paleobiologii PAN, Warszawa, Poland; 9 University of Bucharest, Faculty of Geology and Geophysics, Department of Geology, Bucharest, Romania; 10 University of Dayton, Department of Geology, Dayton, Ohio, United States of America; University of Oxford, United Kingdom

## Abstract

Modern and Cenozoic deep-sea hydrothermal-vent and methane-seep communities are dominated by large tubeworms, bivalves and gastropods. In contrast, many Early Cretaceous seep communities were dominated by the largest Mesozoic rhynchonellid brachiopod, the dimerelloid *Peregrinella*, the paleoecologic and evolutionary traits of which are still poorly understood. We investigated the nature of *Peregrinella* based on 11 occurrences world wide and a literature survey. All *in situ* occurrences of *Peregrinella* were confirmed as methane-seep deposits, supporting the view that *Peregrinella* lived exclusively at methane seeps. Strontium isotope stratigraphy indicates that *Peregrinella* originated in the late Berriasian and disappeared after the early Hauterivian, giving it a geologic range of ca. 9.0 (+1.45/–0.85) million years. This range is similar to that of rhynchonellid brachiopod genera in general, and in this respect *Peregrinella* differs from seep-inhabiting mollusks, which have, on average, longer geologic ranges than marine mollusks in general. Furthermore, we found that (1) *Peregrinella* grew to larger sizes at passive continental margins than at active margins; (2) it grew to larger sizes at sites with diffusive seepage than at sites with advective fluid flow; (3) despite its commonly huge numerical abundance, its presence had no discernible impact on the diversity of other taxa at seep sites, including infaunal chemosymbiotic bivalves; and (4) neither its appearance nor its extinction coincides with those of other seep-restricted taxa or with global extinction events during the late Mesozoic. A preference of *Peregrinella* for diffusive seepage is inferred from the larger average sizes of *Peregrinella* at sites with more microcrystalline carbonate (micrite) and less seep cements. Because other seep-inhabiting brachiopods occur at sites where such cements are very abundant, we speculate that the various vent- and seep-inhabiting dimerelloid brachiopods since Devonian time may have adapted to these environments in more than one way.

## Introduction

**Figure 1:**
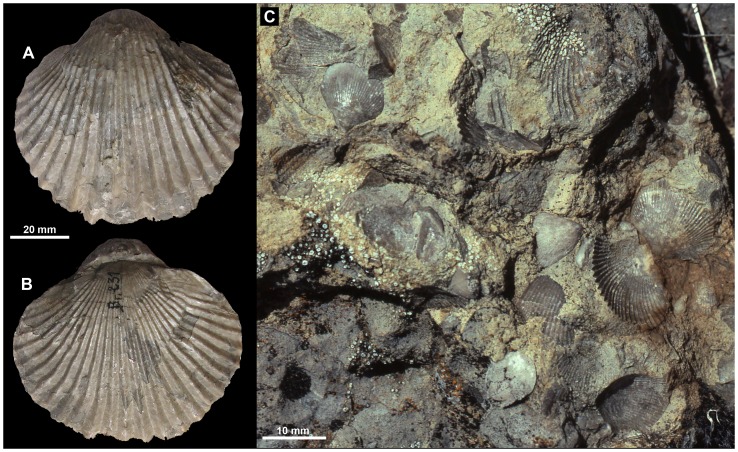
Examples of *Peregrinella*. A, B. *Peregrinella multicarinata* (Lamarck, 1819) from Rottier, southeastern France (EM 36190). C. Field image showing the mass occurrence of *Peregrinella whitneyi* (Gabb, 1866) at the Wilbur Springs seep site in California, USA.

The discovery of highly specialized animal communities around hydrothermal vents and methane seeps in the late 1970s and 1980s [1], [2] and the question of their origin spurred the search for fossil examples of these ecosystems [3]. The major players of the modern, mollusk-dominated fauna, such as vesicomyid bivalves and bathymodiolin mussels, can be traced into the Eocene, and minor bivalve groups and various gastropods range into the Cretaceous [4]–[8]. In contrast, many Paleozoic and Mesozoic seep communities were dominated by organisms that are virtually absent from modern vents and seeps: rhynchonellid brachiopods [9]. Perhaps the most notable of these is the Early Cretaceous genus *Peregrinella* (Figure 1), the largest Mesozoic rhynchonellid brachiopod. *Peregrinella* long puzzled paleontologists because of its large size, its mass occurrence in isolated lenses and its widespread, yet disjunct, distribution [10]–[16].

Early in the 1980s various *Peregrinella* occurrences in the Vocontian basin in southern France were discussed as analogs to modern hydrothermal vent communities [17], [18]. But it was only after the discovery of methane-seep communities in the Gulf of Mexico that *Peregrinella*-bearing deposits were identified as ancient methane seeps [19], based on stable carbon isotope analyses of a Californian example. These authors listed most occurrences of *Peregrinella* and noted that all are found as isolated carbonate lenses within deep-water sediments similar to the Californian example. Therefore they suggested that *Peregrinella* lived exclusively at ancient methane seeps like the major taxa at vents and seeps today [19]. However, brachiopods are rare at modern vents and seeps and are only found in the marginal areas of these habitats. Thus, there are no modern analogs to the large and superabundant *Peregrinella* at fossil methane seeps (Figure 1C) and its paleoecologic and evolutionary traits remain poorly understood. Campbell and Bottjer [19] listed 18 occurrences world-wide, another one was subsequently reported from Alaska [20], and a summary of the biostratigraphic evidence for *Peregrinella* suggested a Berriasian to Hauterivian range for the genus [21].

**Figure 2:**
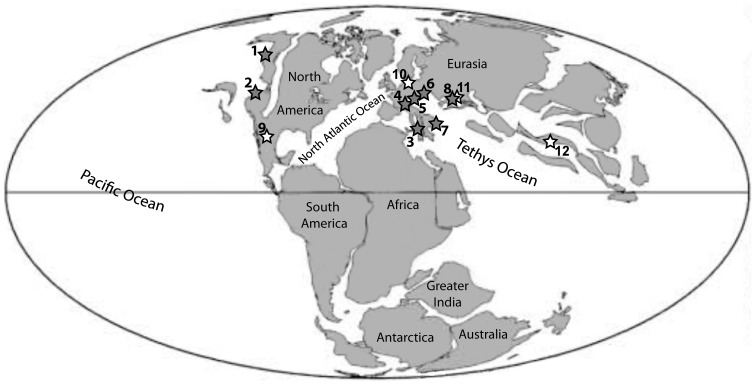
Distribution of the *Peregrinella* occurrences discussed herein, plotted on a paleogeographic map of the late Berriasian (140 m.y.a., [98]). Gray stars indicate occurrences for which new data are available: 1, Bonanaza Creek, Alaska; 2, Rice Valley and Wilbur Springs, California; 3, Incoronata, Italy; 4, Châtillon-en-Dois, Curnier and Rottier, southern France; 5, Musenalp, Switzerland; 6, Raciborsko, Poland; 7, Zizin Valley, Romania; 8, Planerskoje, Crimean peninsula. White stars indicate occurrences known from the literature: 9, Guanajuato seamount, Mexico; 10, Bohrung Werle, northern Germany; 11, Kuban, Russia; 12, Xainza County, Tibet.

Here we investigate strontium-isotope stratigraphy, petrography, stable carbon and oxygen isotopes, and the associated fauna of samples from 11 *Peregrinella* occurrences worldwide (Figure 2) that span its (suggested) stratigraphic range, together with a review of the available literature, to provide new insights into the nature of this enigmatic brachiopod. *Peregrinella* belongs to the superfamily Dimerelloidea, a clade that includes several seep-inhabiting or potentially seep-restricted brachiopods [22]–[27]: therefore, our results may have bearings on the role of brachiopods at vents and seeps throughout most of the Phanerozoic [9].

## Material

Most localities sampled for this work have been described in detail elsewhere; here we just provide essential information and references in which further information can be found. The material is deposited in the Geowissenschaftliches Museum, Georg-August-Universität Göttingen, Germany (GZG), Institute of Paleobiology at the Polish Academy of Sciences, Warsaw, Poland (ZPAL), the collection of the Université Claude Bernard, Lyon-1, France (FSL) and the collection of the Ecole des Mines (EM) that is also housed at the Université Lyon-1, the University of Bucharest, Laboratory of Palaeontology, Romania (LPB), and the Smithsonian Natural History Museum, Washington, DC, USA (USNM).

### Bonanza Creek (Alaska)

*Peregrinella chisania* shells are embedded in a carbonate matrix, a rather unusual lithology for the Chisana Formation that is composed mainly of volcanic rocks and mudstones and is considered as Valanginian-Hauterivian in age [20], [28]. Two small specimens (USNM 487761 and 603602) were available for thin sectioning and isotope analyses (C, O, Sr).

### Châtillon-en-Dois (France)

This locality in the Vocontian Basin is frequently mentioned in the literature on *Peregrinella* and was considered as Hauterivian in age [13], [19], [29], but the original locality has disappeared under urban development. A well-preserved shell was available for isotope analyses (C, O, Sr) from the collection of the Université Claude Bernard, Lyon-1 (FSL 425076).

### Curnier (France)

A limestone block of about 1 m^3^ with *Peregrinella multicarinata* was found as float in a dry creek near Curnier in southeastern France (coordinates: 44°23′23.4″N, 05°15′17.4″E). The associated macrofauna consists of the lucinid bivalve *Tehamatea vocontiana*, the gastropod *Humptulipsia macsotayi* [30], and various ammonites. Samples were collected by SK and LGB in 2010 and the material was used for thin sectioning, isotope analyses (C, O, Sr), and biomarker analysis (GZG.INV.82725-29). This occurrence was considered as Hauterivian in age [19].

### Incoronata (Italy)

Abundant material was collected in 2011 by SK and RG at the base of the Maiolica Formation, which onlaps the flank of the Apulia carbonate platform near the village of Mattinata on the Gargano promontory in southeastern Italy (coordinates: 41°43'25.8"N, 16°02'29.2"E), and was used for thin sectioning and isotope analyses (C, O, Sr; GZG.INV.82745, 46, 54). The locality and its geologic setting have been described elsewhere and the age of the *Peregrinella*-bearing sediments was considered as latest Valanginian-Early Hauterivian (*NC4a* Subzone) [31]–[33].

### Musenalp (Switzerland)

A small block (30×20×20 cm) with abundant *Peregrinella subsilvana* was found about 10 m NE of the Schwändi hut (coordinates 46°56'26.4"N, 8°27'13.5"E) by SK in 2010, and was used for thin sectioning, isotope analyses (C, O, Sr), and biomarker analysis (GZG.INV.82730-38). The scattered *Peregrinella*-bearing blocks on the small and steep pasture around the Schwändi hut are from a nappe of the Préalpes Médianes and are derived from a post-glacial landslide [14]. This site was previously considered as the oldest occurrence of *Peregrinella*, being possibly of late Berriasian age [34].

### Planerskoje (Crimean peninsula)

An isolated carbonate lens with *Peregrinella multicarinata* was found within well-bedded mudstone of the Planerskoje section in the southeastern Crimean peninsula (coordinates: 44°58'08.1"N, 35°12'01.5"E), considered as Hauterivian in age [35], and was sampled by SK in 2007. The petrography, stable isotopes, biomarkers, and fauna of this locality have been described in detail elsewhere [35]–[37]; here we investigate oxygen and strontium isotopes of *Peregrinella* shells (GZG.INV.82739-44).

### Raciborsko (Poland)

Isolated, pyrite-rich carbonate blocks with *Peregrinella multicarinata* occur within dark, marly shales of the Grodziszcze beds in the Silesian unit in the western Carpathians, considered as being of ‘middle Neocomian’ age [13]. Despite various efforts, this locality has not been re-located in recent years but several small specimens of Biernat's collection (ZPAL Bp.III) at the Institute of Paleobiology at the Polish Academy of Sciences, Warsaw, were available for thin sectioning and isotope analyses (C, O, Sr).

### Rice Valley (California)

Isolated limestone lenses with *Peregrinella whitneyi* occur within the Rice Valley outlier of the Great Valley Group in Lake County, California, USA [38]; approximate coordinates: 39°21′02″N, 122°52′01″W). A hand sample collected by KAC was used for thin sectioning, and isotope analyses (C, O, Sr) of *Peregrinella* shells (GZG.INV.82747-52).

### Rottier (France)

*Peregrinella*-bearing limestone crops out near the village of Rottier in southeastern France [29] and were sampled by SK and CG in 2006 and by SK and LGB in 2010, but only a few *Peregrinella* specimens were found. Additional *Peregrinella*-rich material for thin sectioning and isotope analyses (C, O, Sr) was selected from the collection of the Université Claude Bernard, Lyon-1 (FSL 425077-79).

### Wilbur Springs (California)

This is a large, isolated carbonate lens with *Peregrinella whitneyi* enclosed in serpentinite of the Great Valley Group. The petrography, stable isotopes, biomarkers and fauna of this locality have been described in detail elsewhere [39]–[41]. Its age was considered as Valanginian in the older literature but more recently as Hauterivian [19]. A hand sample collected by KAC was used for thin sectioning and for isotope analyses (C, O, Sr) of *Peregrinella* shells (GZG.INV.82753, 82755, 82756); four specimens from the USNM collection were used for stable C and O isotope analyses of *Peregrinella* shells (USNM 603596-603599).

### Zizin Valley (Romania)

Shells of *Peregrinella multicarinata* are found here both within isolated carbonate blocks as well as scattered through the surrounding flysch deposits of the Sinaia Formation, which is reportedly of late Hauterivian to early Barremian age [12], [42]. The *Peregrinella*-bearing carbonates were identified as seep deposits using petrographic, stable isotope, and biomarker investigations [42]. Shells from both types of occurrence were used for isotope analyses (C, O, Sr).

Two shells from active methane seeps in the northern Gulf of Mexico were available for comparative Sr isotope work, one of the bathymodiolin mussel “*Bathymodiolus*” *childressi* collected at brine pool NR-1 (site GC233) in 640 m depth [43], and one of a terebratulid brachiopod, *Ecnomiosa gerda*? Cooper, from Garden Banks block 647 in about 950–1000 m depth [44]. Specimens of a putative *Peregrinella* from a drill core in northeastern Germany [45] were also investigated, but their affinity to *Peregrinella* is questionable.

## Methods

Thin sections of ca. 60 micrometer thickness were produced from the available material and the surfaces of the counterparts were polished to facilitate the selection of sampling sites for isotope analyses. Samples for stable carbon and oxygen isotope analyses were extracted either from the counterparts of the thin sections or from the brachiopod shells using a hand-held microdrill. Carbonate powders were reacted with 100% phosphoric acid at 75°C using a Finnigan Kiel IV Carbonate Device attached to a Finnigan DELTA V PLUS mass spectrometer. All values are reported in per mil relative to the PDB standard by assigning a δ^13^C value of +1.95‰ and a δ^18^O value of −2.20‰ to NBS19. Reproducibility was checked by replicate analysis of laboratory standards and is better than ±0.05‰. Paleotemperatures were calculated from the δ^18^O values of *Peregrinella* shells using the formula provided by [46] and assuming an ice-free ocean with a δ^18^O_seawater_ value of −1.2. [47] and ignoring artifacts that were possibly introduced due to isotope exchange during late diagenesis and rock alteration.

**Figure 3:**
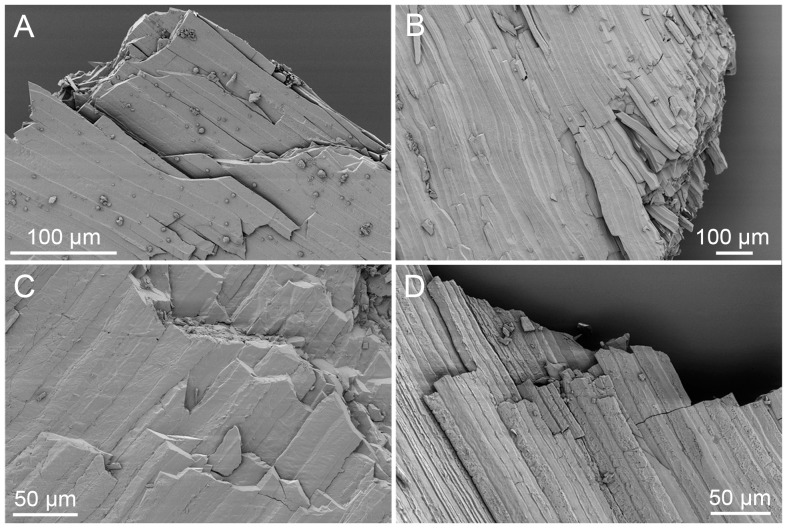
SEM images of well preserved *Peregrinella* shells with unaltered shell microstructure. The examples are from: A: Incoronata (GZG.INV.82745); B: Planerskoje (GZG.INV.82739); C: Musenalp (GZG.INV.82737); D: Curnier (FSL 425078).

For strontium isotopes we analyzed two different *Peregrinella* shells (where available) from each locality listed in the ‘Material’ section to check for potential inhomogeneity, distributed alteration or recrystallization of the original calcite. The microstructure of shell fragments was examined optically and with scanning electron microscopy (LEO 1530 SEM at 3.8 KV; specimens coated with 14 nm of platinum: Figure 3) to check for possible diagenetic alteration or intergrowth with siliciclastic material. Fragments devoid of any indications of recrystallization or contamination were selected and cleaned in distilled water and in pure ethanol. Wherever feasible we also cleaned the fragments by rinsing them in 1 N HCl for about 5 seconds to remove any secondary calcareous material and contaminants [48]–[50]. After another rinse in ultrapure water the shell fragments were dissolved in 2.5 N HCl. Strontium was isolated and recovered from the solutes by ion exchange using Dowex AG-50 cation exchange resin.

Mass spectrometry analyses of Sr isotopic compositions were carried out at GFZ Potsdam, using a Thermo-Finnigan Triton TIMS (thermal ionization mass spectrometry) instrument. Strontium was analyzed in dynamic multicollection mode. For the duration of the study, the mean value obtained for ^87^Sr/^86^Sr of the NBS standard SRM 987 (now NIST 987) was 0.710261±0.000006 (n = 10, 2σ SD). The ^87^Sr/^86^Sr value for SRM 987 used for construction of the LOWESS dataset for conversion of Sr isotope data into ages is 0.710248 [51], which requires application of a normalization factor of 0.999982 for our Sr isotope ratio data. Calculation of ages and age errors was performed using the ‘Look-Up Table Version 5: 04/13’ [52]. The error intervals calculated for our Sr isotope stratigraphy age data (see Table 1) are based on typical 2σ absolute uncertainties for single-analysis, first-measurement ^87^Sr/^86^Sr values of ±0.000020. This uncertainty is estimated from the overall analytical range of values obtained for the NBS standard SRM 987. The only exception is sample PS1804. Here we used the measured 2σ_m_ uncertainty since it was, because of small sample size, slightly higher than the standard-derived uncertainty. Analytical blanks for Sr are regarded negligible compared to the amounts of sample Sr in each analysis; hence, no blank correction was applied. To check for possible radiogenic in-growth of ^87^Sr after shell deposition, we analyzed a few samples for Rb concentrations using a Thermo-Neptune multicollection ICP-MS instrument (GFZ Potsdam). Rubidium concentrations in the carbonate material are very low, in the range of less than 30 ppb. With typical Sr concentrations of at least several hundred ppm in both recent and fossil shell material (compilation of data in [53], [54]), Rb/Sr ratios are low enough to rule out any significant post-depositional radiogenic in-growth of ^87^Sr since shell growth.

**Table 1:** Sr-isotope data and possible stratigraphic ages derived from the LOWESS 5.0 curve [52].

Sample	^87^Sr/^86^Sr ±2σ_m_ meas.	^87^Sr/^86^Sr norm.	age 1 (±2σ) (in Ma)	age 2 (±2σ) (in Ma)	age 3 (±2σ) (in Ma)
Bonanza Creek, Alaska (USNM 603602)	0.707416+/−0.000028	0.707403	**133.20** (+1.85/−0.95)	**125.40** (+1.10/−1.25)	**108.90** (+1.90/−>6.65
Bonanza Creek, Alaska (USNM 487761)	0.707437+/−0.000017	0.707424	**132.60** (+0.70/−0.60)	**126.10** (+0.80/−0.85)	(**104.05** or **96.65**)
Chatillon, France (FSL 425076)	0.707414+/−0.000005	0.707401	**133.25** (+1.10/−0.65)	**125.30** (+0.90/−0.90)	**109.10** (+1.50/−5.70)
Chatillon, France (FSL 425076)	0.707447+/−0.000005	0.707434	**132.40** (+0.60/−0.80)	**126.40** (+0.80/−0.75)	**approx. 100**
Curnier, France (GZG.INV.82729)	0.707440+/−0.000016	0.707427	**132.55** (+0.65/−0.65)	**126.20** (+0.80/−0.80)	(**103.30** or **97.00**)
Incoronata, Italy (GZG.INV.82745)	0.707365+/−0.000006	0.707352	**136.70** (+1.20/−2.40)	**123.30** (+1.00/−1.40)	**111.25** (+0.80/−0.95)
Incoronata, Italy (GZG.INV.82746)	0.707363+/−0.000006	0.707350	**136.80** (+1.20/−2.20)	**123.20** (+1.05/−1.40)	**111.30** (+0.80/−0.95)
Musenalp, Switzerland (GZG.INV.82737)	0.707271+/−0.000007	0.707258	**141.15** (+1.45/−1.20)	**117.50** (+1.75/−1.15)	**113.50** (+0.65/−0.55)
Musenalp, Switzerland (GZG.INV.82738)	0.707270+/−0.000004	0.707257	**141.20** (+1.45/−1.20)	**117.45** (+1.75/−1.15)	**113.50** (+0.65/−0.55)
Planerskoje, Crimea (GZG.INV.82739)	0.707298+/−0.000005	0.707285	**139.75** (+1.25/−1.05)	**119.10** (+1.60/−1.85)	**113.00** (+0.60/−0.65)
Planerskoje, Crimea (GZG.INV.82740)	0.707293+/−0.000007	0.707280	**140.00** (+1.30/−1.05)	**118.75** (+1.65/−1.65)	**113.10** (+0.60/−0.65)
Raciborsko, Poland (ZPAL Bp.III)	0.707399+/−0.000007	0.707386	**133.70** (+2.25/−0.70)	**124.70** (+1.00/−0.90)	**110.05** (+1.05/−4.70)
Rice Valley, California (GZG.INV.82747)	0.707387+/−0.000007	0.707374	**134.65** (+2.15/−1.30)	**124.25** (+0.95/−1.00)	**110.55** (+0.90/−1.55)
Rottier, France (FSL 425077)	0.707454+/−0.000008	0.707441	**132.20** (+0.60/−0.85)	**126.65** (+0.85/−0.75)	
Wilbur Springs, California (GZG.INV. 82755)	0.707389+/−0.000013	0.707376	**134.40** (+2.30/−1.10)	**124.30** (+0.95/−0.95)	**110.45** (+0.95/−1.70)
Wilbur Springs, California (GZG.INV. 82756)	0.707417+/−0.000009	0.707404	**133.20** (+0.95/−0.65)	**125.45** (+0.80/−0.95)	**108.80** (+1.70/−6.00)
Zizin Valley, Romania (LPB III br 387)	0.707392+/−0.000006	0.707379	**134.15** (+2.35/−0.95)	**124.45** (+0.95/−0.95)	**110.35** (+0.95/−2.25)
Zizin Valley, Romania (LPB III br 385)	0.707399+/−0.000005	0.707386	**133.70** (+2.25/−0.70)	**124.70** (+1.00/−0.90)	**110.05** (+1.05/−4.70)
“*Bathymodiolus*” *childressi*, Gulf of Mexico (GZG.INV.82764)	0.709189+/−0.000004	0.709176	**0.00** (+0.65/−0.00)		
*Ecnomiosa gerda*?, Gulf of Mexico (GZG.INV.82765)	0.709170+/−0.000008	0.709157	**0.55** (+0.45/−0.55)		
*Ecnomiosa gerda*?, Gulf of Mexico	0.709181+/−0.000004	0.709168	**0.24** (+0.58/−0.24)		

**Figure 4:**
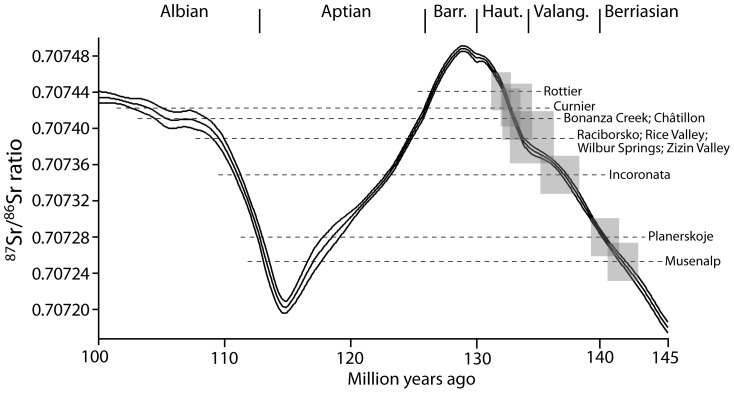
Strontium isotope curve for the Early Cretaceous and its upper and lower confidence interval [52]. Dashed lines indicate our measurements on *Peregrinella* shells; shaded areas indicate the error margins (vertical: Sr isotope ratio; horizontal: geologic age) of those ages that fall within the biostratigraphically possible age range.

‘age 1’ are the ages that fall within the biostratigraphically possible age range, as also shown on Figure 4.

For biomarker analysis, limestone samples from Curnier and Musenalp were prepared and decalcified after a method described previously [40]. After saponification with 6% potassium hydroxide in methanol, the samples were extracted with a microwave extraction system (CEM Discovery) at 80°C and a maximum of 250 W with a mixture of dichloromethane:methanol (3∶1). The resulting total lipid extracts were pre-separated into an *n*-hexane soluble and dichloromethane soluble fraction. For further separation of the *n*-hexane fraction by column chromatography, the samples were separated into four fractions of increasing polarity [55]. Only the hydrocarbon fractions were found to contain indigenous compounds. The polar fractions did not yield genuine lipid biomarker patterns, and were found to be severely affected by thermal maturation and biodegradation. Hydrocarbon fractions were analyzed by coupled gas chromatography- mass spectrometry (GC-MS) with an Agilent 7890 A GC system coupled to an Agilent 5975 C inert MSD mass spectrometer at the Department of Geodynamics and Sedimentology, University of Vienna. The GC-MS was equipped with a 30 m HP-5 MS UI fused silica capillary column (0.25 mm i.d., 0.25 µm film thickness). The carrier gas was helium. The GC temperature program was as follows: 60°C (1 min); from 60°C to 150°C at 10°C/min; from 150°C to 320°C at 4°C/min, 25 min isothermal. Identification of compounds was based on retention times and published mass spectral data. Compound-specific carbon isotope analysis was performed with a Thermo Fisher Trace GC Ultra connected via a Thermo Fisher GC Isolink interface to a Thermo Fisher Delta V Advantage spectrometer at the Department of Terrestrial Ecosystem Research, University of Vienna. Conditions for gas chromatography were the same as described above. Stable carbon isotopic compositions are given as δ values in per mil relative to the PDB standard. Each measurement was calibrated using several pulses of carbon dioxide with known isotopic composition at the beginning and the end of the run. Instrument precision was checked with a mixture of *n*-alkanes (C_14_ to C_38_) of known isotopic composition. Analytical standard deviation was below 0.9‰.

Statistic analyses of faunal data were conducted using the software package PAST v. 2.13 [56].

## Sr-Isotope Stratigraphy of *Peregrinella* Occurrences

Using strontium isotope ratios to correlate the Sr isotopic signature of marine fossils with the global Sr seawater evolution curve [52], we dated the available *Peregrinella* occurrences. As outlined above, the sample preparation procedure was such that alteration or contamination of the analyzed samples is highly unlikely, so that the ^87^Sr/^86^Sr ratios obtained herein can reliably be converted into Sr isotope stratigraphic ages. Reliability of the calculated ages is also supported by the observation that pairs of two independent analyses of different shells from one locality in all cases revealed consistent results (Table 1).

Because all known occurrences of *Peregrinella* are Early Cretaceous (145.0 to 100.5 Ma) in age, we focused on the Early Cretaceous Sr seawater evolution curve. For the Early Cretaceous the Sr seawater evolution curve displays two maxima and one marked minimum (Figure 4), therefore, a specific Sr isotopic composition may correspond to different stratigraphic ages, and age determinations may be ambiguous. To resolve this ambiguity, we used available biostratigraphic and geologic information from the individual *Peregrinella* occurrences to constrain the definite Sr isotope stratigraphic ages. Results, including Sr isotopic compositions of specific samples and their corresponding ages, are presented in Table 1. For comparison, we also present Sr isotopic data on Recent shells of a terebratulid brachiopod and of the mussel “*Bathymodiolus*” *childressi* from methane seeps in the Gulf of Mexico, which are, as expected, indistinguishable from today's Sr seawater composition (^87^Sr/^86^Sr  = 0.709175, [52]). This is important because the Sr-isotope signature of the seeping fluids at these sites often differs substantially from that of seawater [57], [58] and it shows that mollusks and brachiopods incorporate the seawater Sr isotope signature in their shells, not that of the seeping fluids. Therefore we assume that the Sr-isotope signature in the shells of *Peregrinella* reflects that of the seawater in which they lived.

The Sr-isotope ages of two of the fossil sites that are biostratigraphically well-dated are in agreement with biostratigraphy. In particular, the occurrence at the Swiss Musenalp was dated as late Berriasian using tintinnids (calpionellids) [34], consistent with its Sr-isotope age [141.20 (+1.45/−1.20) Ma]; the occurrence at Rottier, France, was dated as early Hauterivian (Jeannoti-zone) using ammonites [29], consistent with its Sr-isotope age [132.20 (+0.60/−0.85) Ma] (Table 1).

Of interest is the Sr-isotope age of the Romanian occurrence in the Sinaia Formation. *Peregrinella* shells from this formation were transported, deriving from turbidites or isolated, allochthonous blocks of limestone enclosed in deep-water strata. These *Peregrinella*-bearing rocks were considered the geologically youngest occurrence, ranging into the early Barremian. This inference, however, was based on foraminifera from the surrounding mudstone of the Sinaia Formation, not from the *Peregrinella* limestone itself. Both investigated shells from this site, one from a carbonate, the other from a siltstone, yielded Sr-isotope values of 0.707379 to 0.707386, indicating a latest Valanginian to earliest Hauterivian age [133.70 (+2.25/−0.70) to 134.15 (+2.35/−0.95) Ma]. Therefore, it seems likely that the Romanian *Peregrinella* shells were eroded from their original latest Valanginian to earliest Hauterivian sediments and reworked into the late Hauterivian-early Barremian Sinaia Formation. The two younger ages that could also be derived from the measured Sr-isotope ratios (123 Ma, early Aptian, and 109 Ma, mid-Albian; see Table 1) can be ruled out because they are younger than the biostratigraphic age of the Sinaia Formation.

The Californian *Peregrinella whitneyi* at Wilbur Springs was considered as Valanginian in the earlier literature [13], [21], [59], [60], but more recently a Hauterivian age was suggested due to the position of the locality just above the last occurrence of the index fossil *Buchia pacifica* [19]. Our Sr isotope data indicate a latest Valanginian to earliest Hauterivian age [133.20 (+0.95/−0.65) to 134.40 (+2.30/−1.10) Ma].

A Hauterivian age was suggested for the occurrence in Rice Valley, California, based on the presence of *Peregrinella whitneyi* and a mollusk assemblage that is now known from many Early Cretaceous seep deposits in California [38]. The Sr-isotope age of this locality [134.65 (+2.15/−1.30) Ma (Table 1)] indicates a latest Valanginian age, similar to the Wilbur Springs site mentioned above. The sediments directly overlying the *Peregrinella*-bearing unit at Rice Valley are dated as Albian-Cenomanian based on palynomorphs [38], thus ruling out the equally consistent Albian Sr-isotope age (Table 1). However, we cannot entirely discard the equally consistent early Aptian Sr-isotope age, but the Hauterivian age is more likely because two of the mollusk species present in the Rice Valley seep carbonate (*Paskentana paskentaensis* and *Retiskenea*? *tuberculata*) are known from other Valanginian-Hauterivian seep deposits, but an Aptian seep deposit from this area (Cold Fork of Cottonwood Creek) contains a different set of taxa [41], [61].

The Polish *Peregrinella* site at Raciborsko is situated roughly at the boundary between the Grodziszcze and Wierzowice (Verovice) shales in the Silesian Nappe and was considered as ‘Neocomian’ [13]. The age of this boundary is diachronous, being oldest (Hauterivian) in the west and becoming younger (as young as Aptian) in the east [62]. Raciborsko is located in the western part of the Silesian Nappe, and our Sr isotope data indicate a basal Hauterivian age for this locality [133.70 (+2.25/−0.70) Ma], consistent with its geographic location.

The Alaskan species *Peregrinella chisania* was previously considered as Valanginian-Hauterivian [20]; the Sr isotope data indicate an early Hauterivian age [133.20 (+1.85/−0.95) to 132.60 (+0.70/−0.60) Ma]. Because the Chisana Formation, from which the species name *P*. *chisania* is derived, hosts Valanginian to Barremian bivalves [28], the equally possible younger (early Aptian and Albian, see Table 1) Sr-isotope ages can be ruled out.

In summary, our Sr-isotope stratigraphic investigation confirms the so far oldest and youngest biostratigraphically well-dated occurrences as late Berriasian and early Hauterivian, respectively. It also shows that some occurrences of *Peregrinella* are slightly older than previously considered, but still within the range late Berriasian–late Hauterivian. Furthermore, it is evident that *Peregrinella* shells and even *Peregrinella*–bearing carbonate blocks can be reworked from their original sediment into younger sediments. Finally, we can refine ages of previously poorly dated occurrences (those dated as Valanginian-Hauterivian or ‘Neocomian’), and show that they are confined within the range late Berriasian–late Hauterivian. An occurrence of *Peregrinella* possibly as young as Albian was reported from sediments of the Guanajuato seamount, central Mexico, an age that was based on co-occurring, reworked fossils of ‘Neocomian’ to Albian age [63]. However, considering that all specimens investigated here are of ‘Neocomian’ age and that the Romanian example documented here indicates that *Peregrinella* can be reworked into younger sediments, it seems likely that also the Mexican occurrence falls within the stratigraphic interval of the other localities, rather than being of Albian age. Thus, *Peregrinella* originated in the late Berriasian and most likely became extinct at the end of the early Hauterivian, giving it a geologic range of 9.0 (+1.45/−0.85) million years according to the Gradstein et al. 2012 time scale [64].

## The Habitats of *Peregrinella*

**Figure 5:**
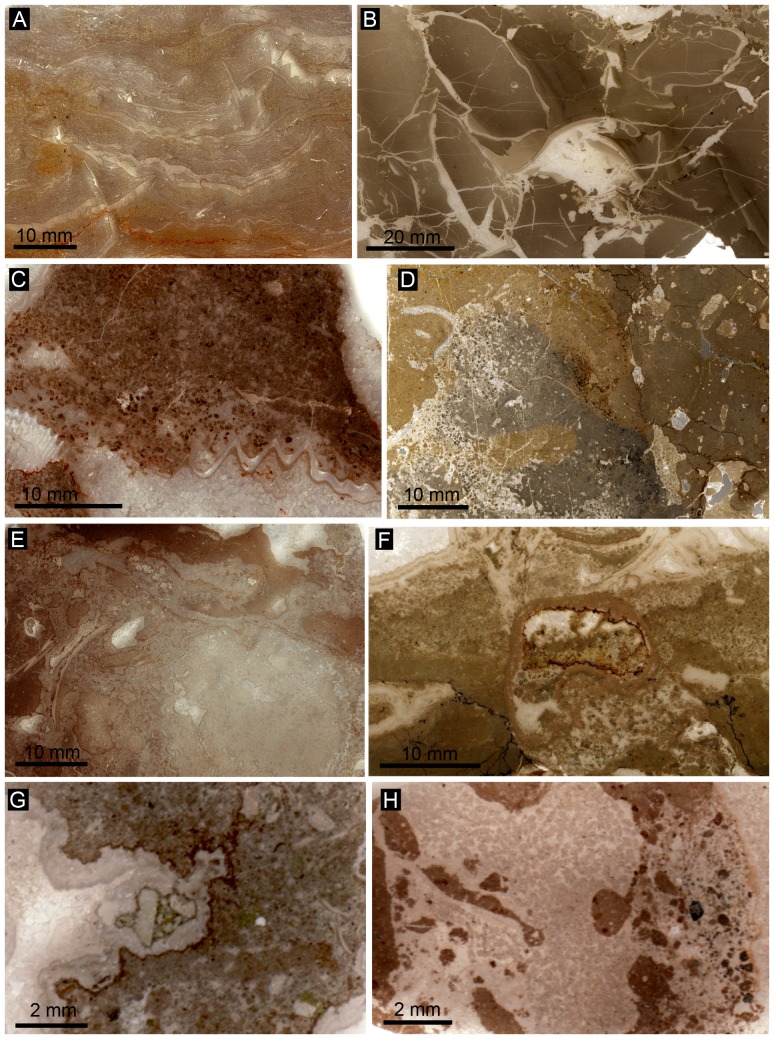
Petrography of *Peregrinella* limestones. Images are ordered by an increasing proportion of seep cements (light colored), compared to microcrystalline carbonate (micrite; dark colored). A: Incoronata (GZG.INV.82754); B: Musenalp (GZG.INV.82734); C: Rottier (FSL 425077); D: Curnier (GZG.INV.82728); E: Rice Valley (GZG.INV.82748); F: Wilbur Springs (GZG.INV.82753); G: Bonanza Creek (USNM 603602), H: Raciborsko (ZPAL Bp.III).

**Figure 6:**
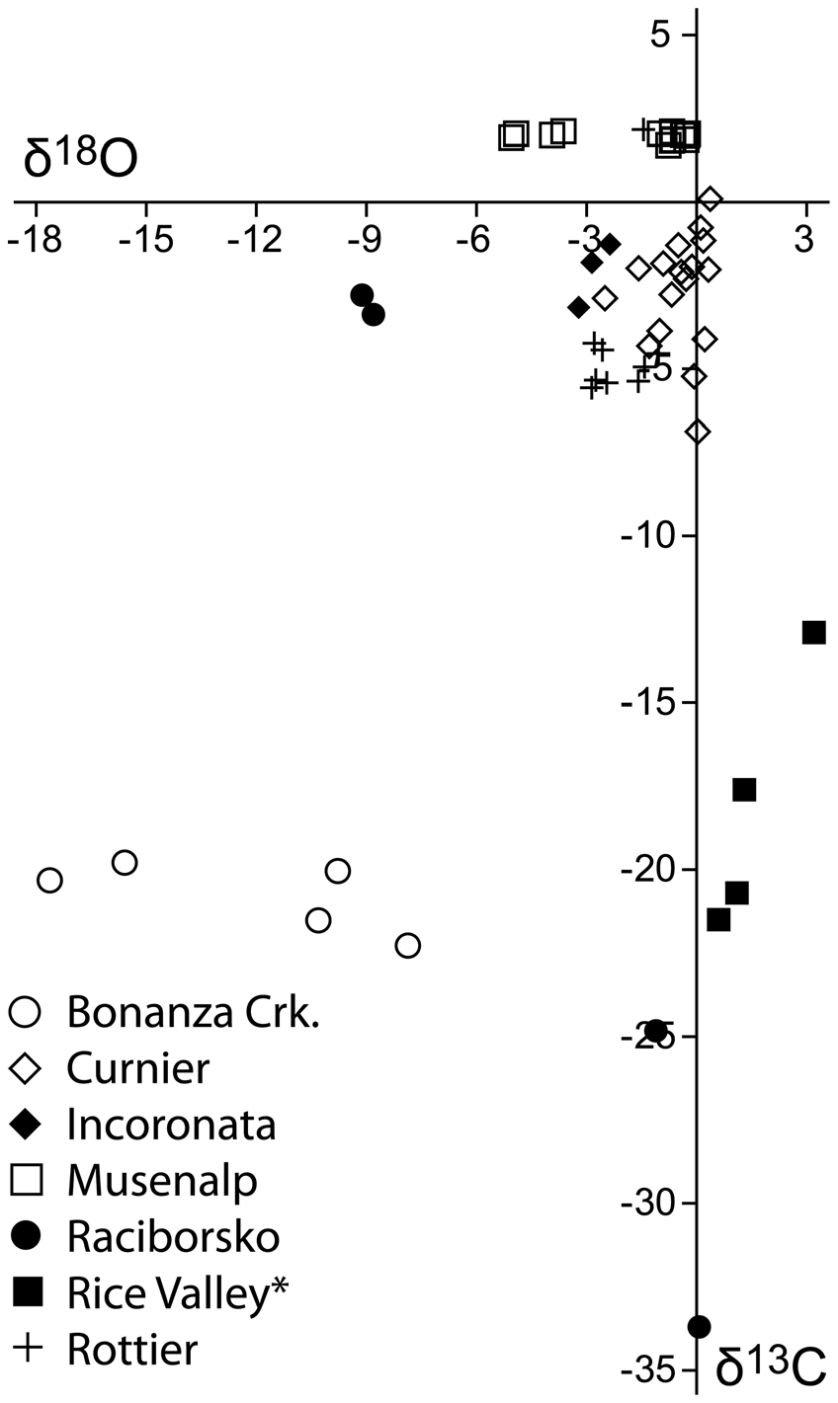
Carbon and oxygen isotope values of *Peregrinella*-bearing limestones. All values are reported relative to the PDB standard. * Data from [99].

To date, three *Peregrinella*-bearing limestones have unequivocally been identified as ancient methane-seep deposits using a combination of petrographic, stable isotope, and biomarker analyses [19], [35], [37], [40], [42]. Appropriate material to carry out at least some of these analyses was available from a further seven localities: Bonanza Creek, Curnier, Incoronata, Musenalp, Raciborsko, Rice Valley, and Rottier. Characteristic petrographic features of seep carbonates are authigenic, early diagenetic carbonate phases, such as microcrystalline calcite (micrite), yellow calcite, and banded and botryoidal aggregates of fibrous cement [39], and the carbon isotope signature of these early diagenetic carbonate phases typically have very negative δ^13^C signatures, significantly lower than marine bicarbonate and occasionally even lower than −50‰ [65]. A detailed description and discussion of the petrographic and stable isotope data of each locality would exceed the scope of the present paper. Therefore we provide a summary of the characteristics of the investigated sites (Table 2) and discuss their implications for reconstruction of the paleoenvironmental conditions under which *Peregrinella* lived. Representative thin section images are shown in Figure 5, and the stable isotope data are summarized in Table 3A and plotted in Figure 6.

**Table 2:** Summary of ecologic and environmental characteristics of *Peregrinella*-bearing localities, compiled from our own data and the literature.

Locality	Species	Max. size (mm)	Type of tectonic margin	Range of δ^13^C-values [‰]* ^1^	Seep cement (%)	Mean δ^18^O-paleotemperature (°C)	References
Bonanza Creek, Alaska	*P. chisania*	30	active	−22.3 to −19.8	∼20	nd	[20] and herein
Yongzhu bridge	*P. donqoensis, P. baingoinensis, P. cheboensis*	49* ^2^	active	nd	nd	nd	[21]
Rice Valley, USA	*P. whitneyi*	40	active	−21.5 to −12.9	∼20	12.2	[99] and herein
Wilbur Springs, USA	*P. whitneyi*	60	active	−24.3 to −19.3	10	13.3	[39] and herein
E of Lhasa, China	*P. gongboxueensis*	62.8	active	nd	nd	nd	[21]
Koniakov Castle, Czech Republic	*P. multicarinata*	26	passive	nd	nd	nd	[11]
Musenalp, Switzerland	*P. subsilvana*	56	passive	1.7 to 2.1	0	17.3	[14] and herein
Raciborsko, Poland	*P. multicarinata*	59	passive	−33.7 to −2.8	<10	nd	[13] and herein
Curnier, France	*P. multicarinata*	70	passive	−6.9 to 0.1	0	14	herein
Zizin Valley, Romania	*P. multicarinata*	85	passive	−29.7 to −20.4	5	12.2	[42]
Incoronata, Italy	*P. garganica*	85	passive	−3.1 to −1.2	0	nd	[32] and herein
Chatillon, France	*P. multicarinata*	85	passive	nd	nd	15.1	herein
Rottier, France	*P. multicarinata*	88	passive	−5.6 to 2.2	0	13.8	[29] and herein
Planerskoje, Crimea	*P. multicarinata*	90	passive	−13.6 to 2.9	<5	11.7	[35]
Koniakov, Czech Republic	*P. silesica*	90	passive	nd	nd	nd	[11]
Kuban, Russia	*P. pinguis*	102	passive	nd	nd	nd	[102]

nd = no data,

*^1^: only the values of carbonate mineral phases are given here, not those of *Peregrinella* shells;

*^2^: size of largest species.

**Table 3:** Summary of the stable carbon and oxygen isotope data.

A - Limestone samples			B - *Peregrinella* shells			
Sample	δ^13^C(PDB) [‰]	δ^18^O(PDB) [‰]	Sample	δ^13^C (PDB) [‰]	δ^18^O (PDB) [‰]	Paleotemperature [°C]
Bonanza Creek, micrite	−19.8	−15.6	Châtillon, FSL 425076	0.0	−0.5	**14**
Bonanza Creek, micrite	−20.3	−17.6	Châtillon, FSL 425076	−0.1	−0.9	**16**
Bonanza Creek, rim cement	−20.0	−9.8	Châtillon, FSL 425076	0.1	−0.6	**14**
Bonanza Creek, rim cement	−22.3	−7.9	Châtillon, FSL 425076	0.2	−1.0	**16**
Bonanza Creek, rim cement	−21.5	−10.3	Curnier, shell 1, GZG.INV.82725	0.5	−0.3	**13**
Curnier, micrite, GZG.INV.82728	−4.3	−1.3	Curnier, shell 1, GZG.INV.82725	0.7	−0.2	**13**
Curnier, micrite, GZG.INV.82728	−3.9	−1.0	Curnier, shell 2, GZG.INV.82726	−0.3	−1.6	**18**
Curnier, micrite, GZG.INV.82728	−2.8	−0.7	Curnier, shell 2, GZG.INV.82726	−0.3	−1.1	**17**
Curnier, micrite, GZG.INV.82728	−2.0	−1.6	Curnier, shell 3, GZG.INV.82727	0.3	0.1	**12**
Curnier, micrite, GZG.INV.82728	−1.3	−0.5	Curnier, shell 3, GZG.INV.82727	0.9	0.2	**11**
Curnier, micrite, GZG.INV.82728	−6.9	0.0	Musenalp, shell 1, GZG.INV.82735	0.8	−1.3	**17**
Curnier, micrite, GZG.INV.82728	−5.2	−0.1	Musenalp, shell 2, GZG.INV.82736	1.1	−1.7	**19**
Curnier, micrite, GZG.INV.82728	−2.9	−2.5	Musenalp, shell 3, GZG.INV.82730	0.4	−1.1	**16**
Curnier, micrite, GZG.INV.82728	−1.8	−0.9	Musenalp, shell 4, GZG.INV.82731	1.1	−0.8	**15**
Curnier, micrite, GZG.INV.82728	−2.0	0.3	Musenalp, shell 5, GZG.INV.82732	1.0	−1.3	**17**
Curnier, micrite, GZG.INV.82728	−1.9	−0.1	Musenalp, shell 5, GZG.INV.82732	0.9	−1.7	**19**
Curnier, micrite, GZG.INV.82728	−4.1	0.2	Planerskoje, shell 1, GZG.INV.82739	−1.4	0.1	**11**
Curnier, micrite, GZG.INV.82728	−2.3	−0.3	Planerskoje, shell 2, GZG.INV.82740	−0.1	0.1	**12**
Curnier, micrite, GZG.INV.82728	−2.1	−0.4	Planerskoje, shell 3, GZG.INV.82741	−0.2	0.0	**12**
Curnier, rim micrite, GZG.INV.82728	0.1	0.4	Planerskoje, shell 4, GZG.INV.82742	−0.8	0.1	**12**
Curnier, rim micrite, GZG.INV.82728	−1.2	0.2	Planerskoje, shell 5, GZG.INV.82743	−0.8	0.0	**12**
Curnier, rim micrite, GZG.INV.82728	−0.8	0.1	Planerskoje, shell 6, GZG.INV.82744	−2.5	0.0	**12**
Incoronata, Italy, GZG.INV.82754	−3.2	−3.3	Rice Valley, GZG.INV.82748	−1.7	−0.5	**14**
Incoronata, Italy, GZG.INV.82754	−1.8	−2.9	Rice Valley, GZG.INV.82749	−3.0	−0.3	**13**
Incoronata, Italy, GZG.INV.82754	−1.3	−2.4	Rice Valley, GZG.INV.82750	−0.6	−0.1	**12.**
Musenalp, micrite, GZG.INV.82733	2.1	−4.9	Rice Valley, GZG.INV.82751	0.8	0.2	**11**
Musenalp, micrite, GZG.INV.82733	1.9	−5.1	Rice Valley, GZG.INV.82752	−2.8	0.4	**10**
Musenalp, micrite, GZG.INV.82733	1.7	−0.8	Rottier, FSL 425077	0.2	−0.2	**13**
Musenalp, micrite, GZG.INV.82733	2.1	−0.2	Rottier, FSL 425077	0.0	−0.3	**13**
Musenalp, micrite, GZG.INV.82733	2.0	−0.4	Rottier, FSL 425078	0.3	−1.3	**18**
Musenalp, micrite, GZG.INV.82733	2.0	−3.9	Rottier, FSL 425078	−2.6	−1.2	**17**
Musenalp, micrite, GZG.INV.82733	2.1	−3.6	Rottier, FSL 425079	−0.1	0.1	**12**
Musenalp, micrite, GZG.INV.82733	1.9	−0.7	Rottier, FSL 425079	0.0	0.2	**11**
Musenalp, micrite, GZG.INV.82733	2.0	−0.3	Wilbur Springs, GZG.INV.82753	−1.1	−0.4	**13**
Musenalp, sparitic rim in shell, GZG.INV.82734	1.9	−0.6	Wilbur Springs, USNM 603596	−1.4	−0.4	**14**
Musenalp, sparitic rim in shell, GZG.INV.82734	1.9	−0.3	Wilbur Springs, USNM 603597	−0.6	−0.2	**13**
Musenalp, sparitic rim in shell, GZG.INV.82734	2.0	−1.0	Wilbur Springs, USNM 603598	−2.5	−1.0	**16**
Musenalp, sparitic rim in shell, GZG.INV.82734	2.1	−0.7	Wilbur Springs, USNM 603599	−0.4	0.2	**11**
Raciborsko, micrite, ZPAL Bp.III	−33.7	0.1	Zizin Valley, LPB III br 364	0.1	0.1	**12**
Raciborsko, peloidal micrite, ZPAL Bp.III	−2.8	−9.1	Zizin Valley, LPB III br 372	0.0	−0.3	**13**
Raciborsko, micrite, ZPAL Bp.III	−24.8	−1.1	Zizin Valley, LPB III br 381	0.3	0.1	**12**
Raciborsko, peloidal micrite, ZPAL Bp.III	−3.4	−8.8	Zizin Valley, LPB III br 381	0.3	0.1	**11**
Rice Valley*	−21.5	0.6	Zizin Valley, LPB III br 389	0.4	−0.4	**13**
Rice Valley*	−12.9	3.2				
Rice Valley*	−20.7	1.1				
Rice Valley*	−17.6	1.3				
Rottier, micrite, FSL 425077	2.2	−1.5				
Rottier, micrite, FSL 425077	−5.3	−2.8				
Rottier, micrite, FSL 425077	−5.4	−2.5				
Rottier, micrite, FSL 425077	−5.6	−2.9				
Rottier, micrite, FSL 425077	−5.4	−1.6				
Rottier, micrite, FSL 425077	−4.9	−1.4				
Rottier, micrite, FSL 425077	−4.5	−1.0				
Rottier, rim micrite, FSL 425077	−4.2	−2.8				
Rottier, rim micrite, FSL 425077	−4.4	−2.6				

*Data from [99].

The investigated occurrences of *Peregrinella* in this study fall within three categories:

1) Occurrences where *Peregrinella* has clearly been transported away from its original living place: Incoronata and Musenalp. Both show bedding and alignment of *Peregrinella* shells parallel to bedding (although less obvious in the case of the Musenalp). Both lack typical seep cements (Figures 5A, B), and both have δ^13^C-signatures of marine carbonate (Figure 6). No conclusions can be drawn about the original habitat of *Peregrinella* at these sites. In the case of the Incoronata locality, which overlies the mid-Valanginian drowning surface between the Maiolica pelagic limestones and the underlying coarse-grained slope debrites, *Peregrinella* has been considered as a disaster taxon that bloomed due to the environmental disruptions linked to the late Weissert Oceanic Anoxic Event [33], [66].

2) *In situ* occurrences within carbonate settings with only minor ^13^C depletion: Curnier and Rottier. The limestones of both sites show evidence for bioturbation, they lack typical seep cements, but authigenic micrites are present (Figures 5C, D). Their carbon isotope signatures (as low as −6.9 and −5.6‰, respectively, Figures 6, Table 2) are below that of marine carbonate, but clearly not low enough to rule out carbon sources other than methane. However, considering that they were deposited in an carbonate setting, a strong input of marine carbonate is obvious, which would have diluted a potentially much lower original signal [37], [65]. We were not able to reproduce the extremely high δ^13^C values of up to +20‰, as reported earlier from Rottier [18]. The accompanying mollusk fauna at Curnier and Rottier consists of taxa that are otherwise found exclusively at methane-seep deposits [30], [67]. Furthermore, the presence of ^13^C-depleted PMI and phytane in the Curnier limestone (see below) indicates that anaerobic oxidation of methane occurred in the depositional environment [65], agreeing with methane seepage. The *Peregrinella*-bearing limestones at Curnier and Rottier are therefore interpreted as ancient methane-seep deposits, probably reflecting diffuse seepage sites.

3) *In situ* occurrences in carbonate bodies with ^13^C-depleted early diagenetic cements: Bonanza Creek, Raciborsko, and Rice Valley. Authigenic micrites, and typical seep cement such as banded and botryoidal cement are common in these limestones (Figures 5F-H, Table 2). The carbon isotope signatures of these cements are as low as −33.7 to −21.5‰ (Figure 6, Table 2) and are thus well within the range of other *Peregrinella*-bearing seep limestones [19], [35], [42]. The associated fauna, where present, consists largely of seep-endemic mollusks [41]. Therefore, Bonanza Creek, Raciborsko, and Rice Valley are here also identified as ancient seep deposits.

**Figure 7:**
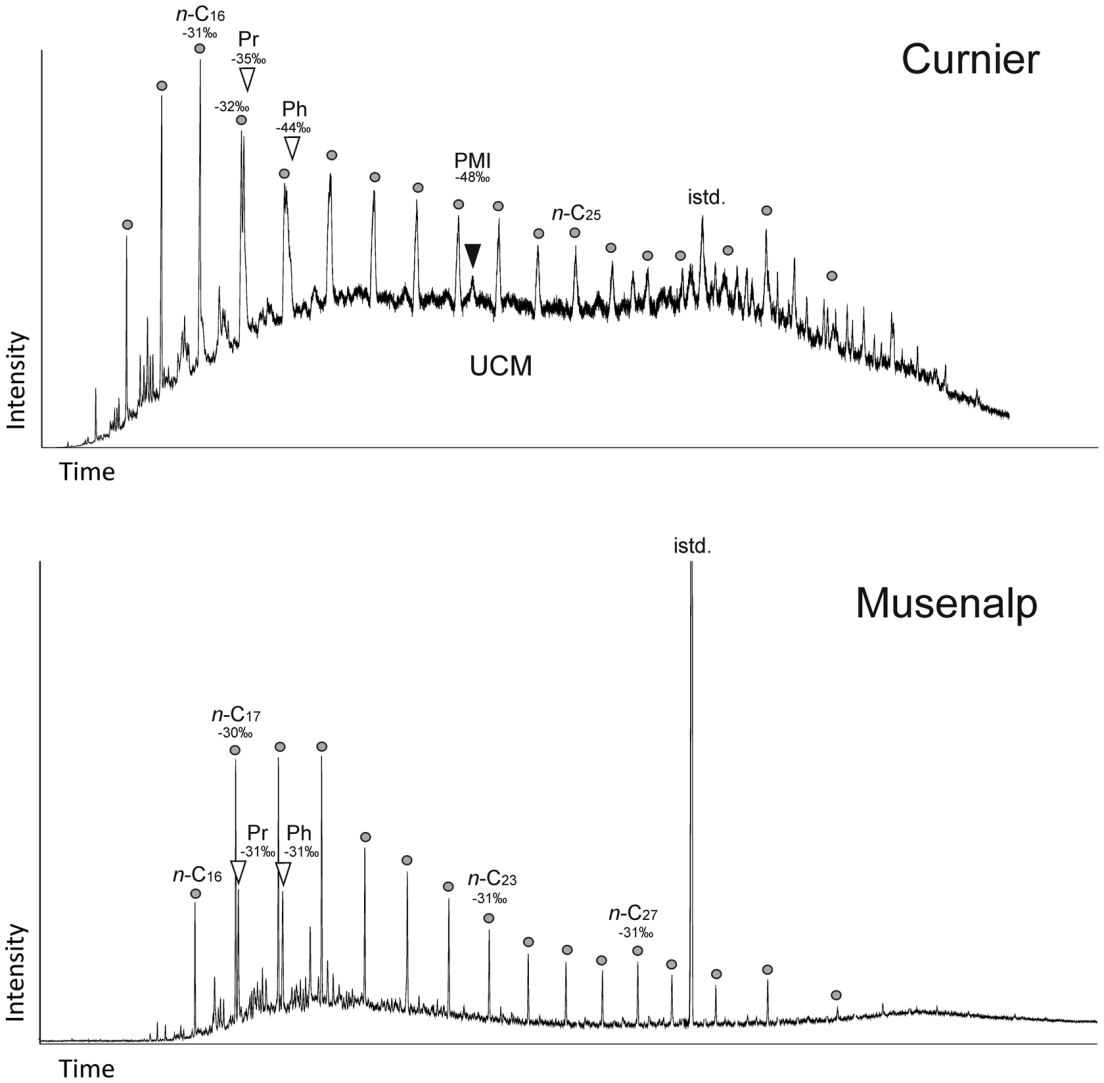
Hydrocarbon fractions (total ion currents) of the Curnier and Musenalp limestones with *Peregrinella*. Circles: *n*-alkanes; white triangles: regular isoprenoids; black triangles: irregular isoprenoids; Pr: pristane; Ph: phytane; PMI: pentamethylicosane; istd.: internal standard; UCM: unresolved complex mixture; compound-specific δ^13^C values are indicated in per mil relative to the Peedee belemnite standard.

Lipid biomarker data have been reported for *Peregrinella*-bearing seep limestones from the Wilbur Springs [40], Planerskoje [37], and Zizin Valley [42] localities. For this study two new limestone samples suited for biomarker analyses were available. The hydrocarbon fraction of the Curnier limestone is typified by an unresolved complex mixture (Figure 7), reflecting a crude oil pattern and indicating pronounced biodegradation. The timing of possible oil ingress and biodegradation cannot be constrained with certainty. Thus, the possibility of syndepositional oil seepage cannot be ruled out. Although the observed compound pattern has obviously been affected by biodegradation and thermal maturity, two compounds and their carbon isotopic compositions point to syndepositional methane seepage, having fueled the anaerobic oxidation of methane involving methanotrophic archaea. Irregular, ^13^C-depleted pentamethylicosane (PMI) is a stable and particularly long-lasting biomarker of methanotrophic archaea [65], [68]. Its δ^13^C value of −48‰ is much higher than values of PMI typically observed at methane seeps; yet, it is also much lower than the values of the regular isoprenoid pristane in the same sample, the precursors of which are produced by phototrophs [69]. Most likely, the relatively high δ^13^C value of PMI is due to masking of its pristine isotopic signature caused by the unresolved complex mixture made up of compounds with higher ^13^C content, on average, than PMI. Hence, it is difficult to determine whether the observed intermediate value of PMI represents a pure methanotrophic or mixed source (methanotrophic and methanogenic archaea) signal. Moreover, the isotopic composition of the regular isoprenoid phytane (−44‰) is much lower than that of pristane (−35‰), revealing that phytane did not exclusively derive from phototrophic organisms. The greater ^13^C-depletion of phytane is best explained by input from methanotrophic archaea (archaeal diethers), in addition to input from phototrophic organisms (chlorophyll), resulting in a carbon isotopic composition that falls between the compositions commonly observed for lipids of methanotrophs and phototrophs. To sum up, in combination with the lowest δ^13^C_carbonate_ values of −6.9‰ found for the Curnier limestone, the observed lipid patterns are best explained by anaerobic oxidation of methane at a seafloor seep. The seepage fluids also may have included crude oil or, alternatively, oil may have intruded the limestone at a later stage. Unlike the Curnier limestone, no biomarker evidence for seepage was found for the Musenalp limestone, agreeing with its marine δ^13^C_carbonate_ values (Figure 6). No irregular isoprenoids were detected in the Musenalp sample, and the regular isoprenoids pristane and phytane as well as *n*-alkanes yielded δ^13^C values (−31 to −30‰) that are typical for lipids resulting from marine primary production in the photic zone [70].

## Impact of *Peregrinella* on Other Seep Inhabitants

The sheer abundance of *Peregrinella* at the localities it colonized (Figure 1) has led to suggestions that it had a negative impact on at least some of the co-occurring taxa, in particular infaunal and semi-infaunal bivalves [19], [35], but these suggestions were based only on observations for individual *Peregrinella* occurrences. We therefore attempted to address this question in a more comparative way. The mollusks in methane-seep communities of the late Mesozoic show an overall similarity in faunal composition, although with a notable turnover during the mid-Cretaceous, and are often dominated by the modiomorphid bivalve *Caspiconcha*, several seep-restricted lucinid bivalves, and the gastropod *Hokkaidoconcha* [8], [30], [71]–[75]. Therefore, our comparisons of specimen size and diversity of the seep mollusk fauna in the presence or absence of *Peregrinella* focused on two time intervals: first the late Jurassic through late Cretaceous (henceforth referred to as the ‘late Mesozoic’), to assess possible impacts of *Peregrinella* on the late Mesozoic seep fauna in general, and second only the time interval spanning the stratigraphic range of *Peregrinella* (henceforth referred to as the ‘*Peregrinella* interval’). The faunal data for these analyses and their sources are summarized in Table 4; included are only sites where we had some confidence that the fauna had been adequately sampled, i.e., reports of *Peregrinella* from collections with unknown sampling strategies (such as Bonanza Creek or Châtillon-en-Dois reported here) are not included.

**Table 4:** *Peregrinella* and chemosymbiotic bivalves at Cretaceous seep deposits.

Locality (age)	*Peregrinella*	Solemyidae, size (mm)	Thyasiridae, size (mm)	Lucinidae, size (mm)	References
**Beauvoisin (Oxfordian)**	**a**			*Beauvoisina carinata*, 200	[103]
**Gateway Pass (Tithonian)**	**a**			lucinid, 50	[104]
**NW Berryessa (Tithonian)**	**a**			*Tehamatea ovalis*	[41]
**Paskenta (Tithonian)**	**a**	*Acharax stantoni*, 58		*Tehamatea ovalis*, 60	[41], [105]
				*Tehamatea colusaensis*, 95	
**Stony Creek (Tithonian)**	**a**	*Acharax stantoni*		*Tehamatea colusaensis*	[41]
				*Tehamatea ovalis*	
* **Sassenfjorden (Berriasian)**	**a**	solemyid, 77	thyasirid, 50	lucinid, 94	[76]
* **Planerskoje (Berriasian)**	**p**			*Tehamatea vocontiana*, 70	[30], [35]
* **Musenalp (Berriasian)**	**p**				[14] and herein
* **Bear Creek (Valanginian)**	**a**	*Acharax stantoni*, 60		*Tehamatea ovalis*, 25	[30], [101]
* **Little Indian Valley (Valanginian)**	**a**	*Solemya* sp.			[41]
* **Rocky Creek (Valanginian)**	**a**	*Acharax stantoni*, 56		*Tehamatea colusaensis*, 145	[30]
* **Raciborsko (Hauterivian)**	**p**				[13] and herein
* **Koniakov (Hauterivian)**	**p**			*Lucina* sp., 22	[11], [106]
* **Koniakover Schloss (Hauterivian)**	**p**			*Lucina valentula*, 13	[11], [106]
				*Lucina obliqua*, 11	
				lucinid sp. ind., 70	
* **Curnier (Hauterivian)**	**p**			*Tehamatea vocontiana*, 50	herein
* **Rottier (Hauterivian)**	**p**			*Tehamatea vocontiana*, 200	[30]
* **Wilbur Springs (Hauterivian)**	**p**	*Acharax stantoni*, 70		*Tehamatea colusaensis*, 77	[30], [105]
**Baska (Barremian)**	**a**			lucinid indet., 30	[106]
**Eagle Creek (Barremian)**	**a**	*Acharax stantoni*, 47		*Tehamatea ovalis*	[101]
**Kuhnpasset Beds (Barremian)**	**a**	*Solemya* sp., 50		*Amanocina kuhnpassetensis*, 129	[107]
**Awanui I (Albian)**	**a**			*Cubathea awanuiensis*, 27	[30], [78]
				*Amanocina raukumara*, 33	
**Awanui II (Albian)**	**a**			*Cubathea awanuiensis*, 50	[30], [78]
**CFCC (Albian)**	**a**	*Acharax stantoni*, 35		*Lucina* sp., 22	[30]
**Ispaster (Albian)**	**a**			*Tehamatea agirrezabalai*, 135	[30], [108]
**Ponbetsu (Albian)**	**a**	*Acharax mikasaensis*, 55	*Thyasira tanabei*, 11.7	"*Nipponothracia*" *ponbetsensis*, 120	[77]
**Awanui GS 688 (Cenomanian)**	**a**		thyasirid, 6	*Amanocina raukumara*, 80	[30], [78]
				*Cubathea awanuiensis*, 48	
				*Ezolucina* ? sp. "ridge", 37	
**Obira-cho (Cenomanian)**	**a**	solemyid indet.	*Thyasira tanabei*, 10.4	lucinid indet., 43	[77]
				*Miltha* sp.	
				*Amanocina yezoensis*, 100	
**Omagari lens (Campanian)**	**a**		*Thyasira tanabei*, 50	lucinid *ponbetsensis*, 60	[77]
				*Tehamatea* sp.?, 50	
**Romero Creek (Campanian)**	**a**	*Solemya* sp.	*Thyasira cretacea*		[41]
**Sada Limestone (Campanian)**	**a**	solemyid, 70	*Thyasira hataii*, 80	*Myrtea*? sp., 35	[30], [109]
				lucinid, 71	
**Waipiro I (Campanian)**	**a**			*Ezolucina* sp., 120	[30], [78]
**Waipiro III (Campanian)**	**a**	solemyid, 20	thyasirid, 10	lucinid, 30	[30], [78]
**Yasukawa (Campanian)**	**a**	*Acharax cretacea*, 60	*Thyasira tanabei*, 10.4	*Miltha* sp., 18	[110]
				*Myrtea* sp., 10	

* = sites of the *Peregrinella* interval, a = absent, p = present.

We performed a set of tests with each of the three infaunal chemosymbiotic bivalve families Solemyidae, Thyasiridae and Lucinidae. First, for the late Mesozoic we tested whether they were more commonly (1) present, and (2) larger, at seep sites outside the *Peregrinella* interval than at seep sites during the *Peregrinella* interval. Second, for the *Peregrinella* interval we tested whether they were (1) more frequently absent from, and (2) smaller at sites with *Peregrinella* than at sites without *Peregrinella*. For the size tests, only the largest record was used when more than one species of a family was present at a locality.

**Figure 8:**
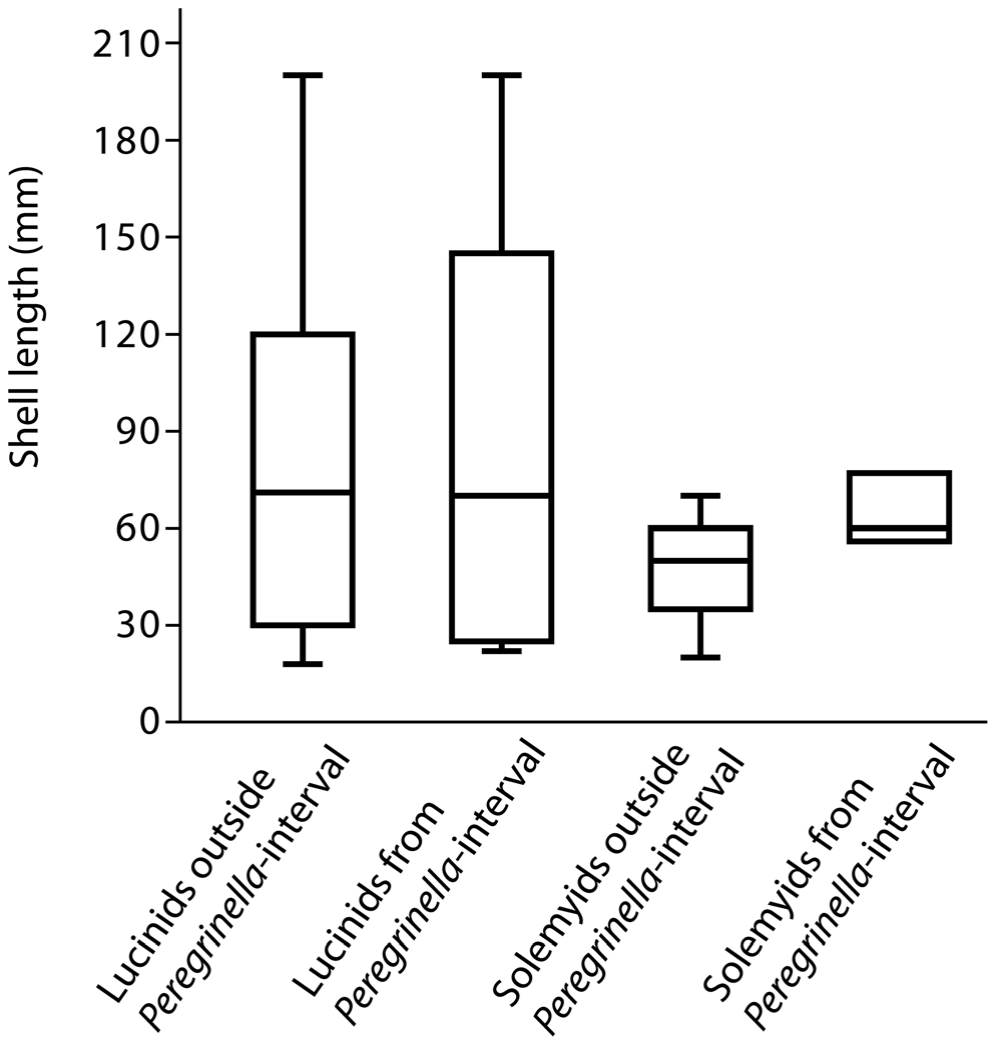
Shell sizes of chemosymbiotic bivalves at late Mesozoic methane seeps.

During the late Mesozoic, all three bivalve families were neither more common nor larger at seep sites outside the *Peregrinella* interval than during the *Peregrinella* interval (Figure 8). However, in the case of the thyasirids these comparisons are problematic: they were reported from shallow-water seeps from the Berriasian on Svalbard [76], are entirely absent from seeps during the remainder of the lower half of the Early Cretaceous, and re-appear in the Albian of Japan and New Zealand [77], [78]. An alternative explanation to a potential impact of *Peregrinella* is that thyasirids did not colonize deep-water seeps until Albian time.

When only the *Peregrinella* interval was considered, lucinids were neither more frequently absent from, nor smaller at sites with *Peregrinella* than at sites without *Peregrinella*. In a rigorous statistical sense this is also true for the solemyids (Fisher's exact test, p = 0.23), although it is remarkable that solemyids occur at all non-*Peregrinella* sites but only at a single site with *Peregrinella* (Table 4). In summary, contrary to previous suggestions, we found little empirical evidence that *Peregrinella* had a negative impact on the presence or size of infaunal bivalves at seeps. However, the observation from the seep deposit at Wilbur Springs that *Peregrinella* is more abundant than the co-occurring modiomorphid bivalves [19] (Figure 1) also applies to the infaunal chemosymbiotic bivalves.

**Figure 9:**
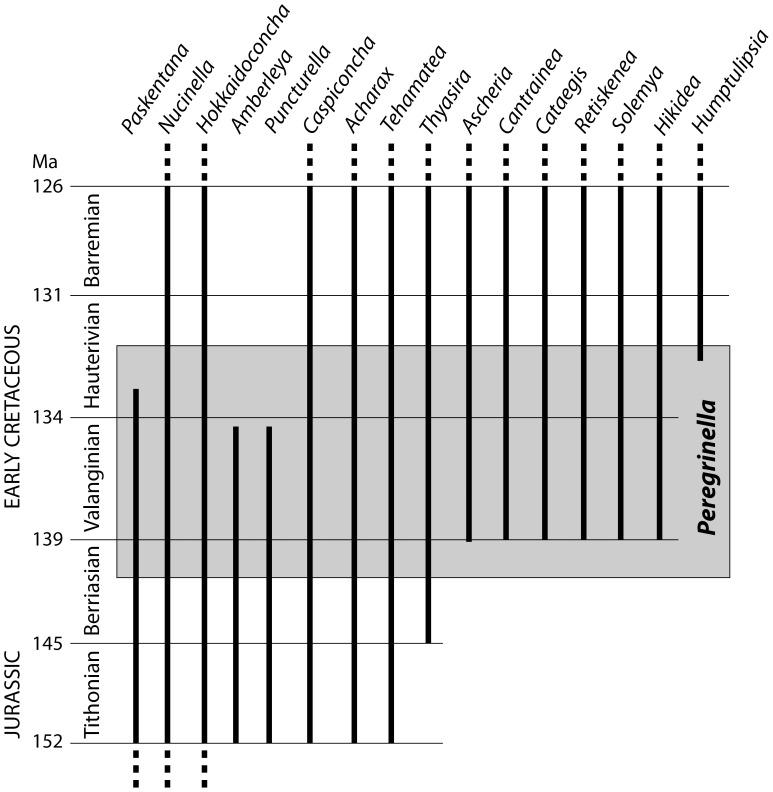
Geologic ranges of seep-inhabiting mollusk genera and *Peregrinella* during the Late Jurassic – Early Cretaceous. Data from [8], [25], [30], [41], [61], [67], [74], [100], [101]; absolute ages from [64].

At localities where *Peregrinella* occurs, it is always more abundant than any other taxon. Therefore, we were interested in whether the presence of *Peregrinella* affected species diversity at the sites it colonized. For the *Peregrinella* interval we tested whether seep sites with fewer than 10 associated species (i.e., excluding *Peregrinella*) are more common among the *Peregrinella*-bearing deposits than seep sites with more than 10 associated species. Sites without associated species were excluded. This analysis showed no significant difference (Fisher's exact test, p = 0.56, Table 5). At its various occurrences, *Peregrinella* coexisted with virtually all coeval seep mollusks, as well as occasional tube worms and sponges. Neither the origin nor the extinction of *Peregrinella* coincides with the origin or extinction of any coeval seep-inhabiting mollusk (Figure 9).

**Table 5:** Numbers of species at seep deposits from the *Peregrinella* interval; a = absent, p = present.

Locality	# associated species	*Peregrinella*	Reference
Musenalp, Switzerland	1	p	[14]
Curnier, France	2	p	herein
Rottier, France	2	p	[29] and herein
East Berryessa, USA	3	a	[41]
Foley Canyon, USA	3	p	[41]
Gravelly Flat, USA	3	p	[41]
Rice Valley, USA	4	p	[41]
West Berryessa, USA	4	a	[41]
Little Indian Valley, USA	5	a	[41]
Koniakov, Czech Republic	5	p	[11], [106]
Planerskoje, Crimean peninsula	7	p	[35]
Wilbur Springs, USA	10	p	[41], [71]
Rocky Creek, USA	13	a	[41], [71], [101]
Koniakov Castle, Czech Republic	17	p	[11], [106]
Bear Creek, USA	18	a	[41], [71], [101]

## Environmental Impacts on *Peregrinella*

*Peregrinella* reached significantly greater maximum sizes at passive continental margins than at active margins (Mann-Whitney *U* test, p = 0.04; Table 2). Assuming that environmental disturbances such as turbidites are more common at active margins than in deep shelf settings at passive margins, it may be inferred that *Peregrinella* was sensitive toward such environmental disturbances.

The intensity of seep fluid flow plays a major role in structuring seep communities today because some taxa prefer sites with focused, advective fluid flow while others prefer sites of slow, diffusive seepage [79]. The apparent restriction of *Peregrinella* to seeps suggest that it benefited, in as-yet unknown ways, from the seeping fluids and the reduced compounds in them. Most likely, *Peregrinella* benefited from chemotrophic bacteria using methane and/or sulfide, but whether *Peregrinella* was filter-feeding on free-living bacterioplankton, for example like modern vent and seep-inhabiting barnacles [80], or had a symbiotic relationship with such bacteria, remains unknown and cannot be resolved with the data presented here. We were interested whether *Peregrinella* had a preference for a certain paleo-seep fluid intensity and compared the maximum size of *Peregrinella* to two proxies for seepage intensity: (i) the carbon isotope signature of the enclosing limestone, and (ii) the abundance of the typical seep cements such as banded or botryoidal cement, quantified based on thin section observations; the localities with uncertain original habitat type, Incoronata and Musenalp, were not used in this comparison. There is no correlation between the maximum size of *Peregrinella* and the carbon isotope signature of the enclosing limestone, but we found a significant negative correlation between the maximum size of *Peregrinella* and the abundance of seep cements (i.e. fibrous, banded and botryoidal cement rather than micrite): *Peregrinella* is on average larger at sites with fewer seep cements (p<0.003, Table 2), suggesting that it was better adapted to diffusive rather than advective seepage [37].

**Figure 10:**
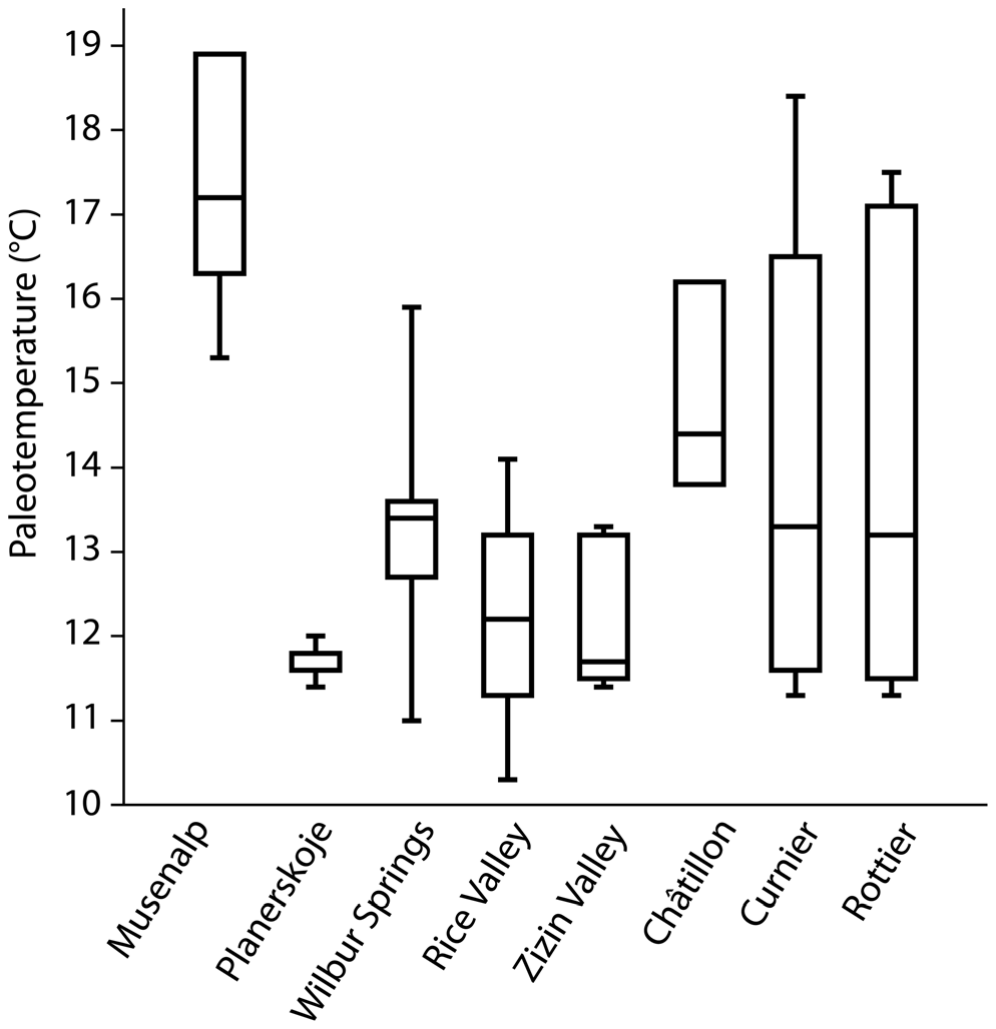
Paleotemperatures derived from δ^18^O values of *Peregrinella* shells. Localities ordered from oldest (left) to youngest (right).

Paleo-temperatures calculated from δ^18^O values of *Peregrinella* shells indicate that these specimens lived at temperatures ranging from 10 to 19°C (Tables 2, 3B). At some localities different shells showed a remarkably uniform range of values (i.e., Planerskoje: 11 to 12°C) while at other localities different shells showed a wide scatter of values (i.e., Rottier: 11 to 18°C; Curnier: 11 to 18°C). The data are summarized in Figure 10. These absolute values should be treated cautiously because some rhynchonellid brachiopods exert vital effects when incorporating oxygen isotopes into their shells [46]. But because we investigated only specimens of a single genus, we infer that all investigated specimens lived within a temperature range spanning about 9°C from the coldest to the warmest. Does this reflect the preferred temperature range of the genus *Peregrinella* in general? Unfortunately, the available material from the northernmost occurrence, the Alaskan Bonanza Creek site, was too sparse for oxygen isotope analyses. Therefore *Peregrinella* might have lived also at lower temperatures than inferred here and thus might have had a broader temperature range than the 9°C estimated from the available specimens.

## Brachiopod Geologic Ranges

The 9 m.y. long geologic range of *Peregrinella* appears short when compared to genera of seep-inhabiting mollusks, including chemosymbiotic bivalves, which may reach geologic ranges of 50 m.y. or more [4], [30]. But how does this compare to the geologic ranges of other brachiopod genera? Virtually all brachiopod genera that dominated vent and seep deposits in the geologic past belong to a single rhynchonellid superfamily, the Dimerelloidea [22], [24]–[27], [81] and their geologic ranges seem to never exceed 10 m.y. in duration (see fig. 9.6 in [23]). To investigate how the geologic ranges of *Peregrinella* and other dimerelloids compare to those of rhynchonellid brachiopod genera in general, we summarized the geologic ranges of genera of the Rhynchonellida listed in Sepkoski's compendium [82], using the following criteria: only taxa with their first appearances (FAs) and last appearances (LAs) given at least to series level were included. Only taxa having their FAs in Devonian through Cretaceous were included. Subgenera and genera with “?” were excluded. Taxa with questionable FAs or LAs, as indicated by a “?” were excluded. Stratigraphic subdivisions such as lower, middle, and upper were ignored. Taxa with FAs or LAs in the Leonardian (Permian) were excluded because this stage could not be correlated with the Gradstein et al. 2012 time scale [64]. The geologic ranges of the remaining 483 genera were calculated as follows: numerical age of base of FA minus numerical age of end of LA. This resulted in a median geologic range of 12.7 m.y., and 25 and 75 percentiles of 6.6 and 17.3 m.y., respectively. Thus the 9 m.y. range of *Peregrinella* and the geologic ranges of dimerelloid genera in general, are somewhat shorter than the median, but certainly not abnormal for rhynchonellid brachiopods. In this respect, dimerelloids differ from mollusk genera at seeps which are, on average, longer-lived than marine mollusks in general [4]. These observations fit with two macroevolutionary patterns among brachiopods and mollusks. First, on low taxonomic levels (e.g., genus, species) brachiopods show higher origination and extinction rates than mollusks [83], [84], as seen here also among their seep-inhabiting members. Second, on the family/superfamily level brachiopods appear to persist longer in deep-water environments than gastropods do [85], consistent with the long persistence of the Dimerelloidea in the vent/seep environment.

## Discussion

The oldest occurrences of *Peregrinella* are the Musenalp site in Switzerland and the Planerskoje site in the eastern Crimean peninsula; both are of late Berriasian age. The genus thus apparently originated in the Tethys Ocean [21], in contrast to a previous hypothesis of an origin in California and subsequent dispersal to Europe [13], [45]. In the Valanginian *Peregrinella* had spread eastward as far as Tibet [21] and by Hauterivian time it had spread to California and Alaska. Whether this spread to the American West coast was eastward or westward from the Tethys remains unclear. The present understanding of Early Cretaceous ocean currents [86] makes a westward spread possible, although an eastward spread along the active continental margins around the North Pacific Ocean also seems plausible [87]. The youngest occurrences are those from southern France and Alaska, and are of late early Hauterivian age.

The timing of the appearance and disappearance of *Peregrinella* is puzzling, because neither does it coincide with the appearance and disappearance of any other seep-inhabiting taxon, nor with any of the major oceanic disturbances such as oceanic anoxic events (OAEs). Its extinction at the end of the early Hauterivian roughly coincides with the ‘Faraoni event’, a presumed, short-lived anoxic event in the late Hauterivian [88], but this event was apparently restricted to the Tethyan Realm and does not explain the disappearance of *Peregrinella* from California and Alaska.

The implied concurrent demise of *Peregrinella* and the large, seep-inhabiting bivalve *Caspiconcha* at Cretaceous seeps in figure 15 of [74] is not supported by our Sr-isotope stratigraphy results and by the finding of a mid-Cenomanian *Caspiconcha*-dominated seep deposit in New Zealand [78]. There are about 36 m.y. between the extinction of *Peregrinella* at the end of the early Hauterivian and the last *Caspiconcha*-dominated seep deposit.

The apparent preference of *Peregrinella* for diffusive seepage rather than for focused, advective fluid flow is surprising considering that taxa dominating modern vents and seeps prefer strong fluid flow and resulting high hydrogen sulfide concentrations [79], [89], [90]. A potential implication of this pattern is that the link of *Peregrinella* to seeps is related to methane rather than to hydrogen sulfide. Because hydrogen sulfide is quickly oxidized in the presence of oxygen, it only reaches the seafloor when fluid flow is strongly advective. When fluid flow is slow and diffusive, hydrogen sulfide is oxidized before it reaches the sediment-water interface, as shown by modeling approaches and *in situ* measurements [91], [92]. Methane, on the other hand, is only oxidized biologically in marine environments and therefore has a greater chance to reach the sediment-water interface under diffusive flow conditions than hydrogen sulfide. In case of the Planerskoje and Zizin Valley seep deposits, the presence of molecular fossils of aerobic methane-oxidizing bacteria [37], [42] reveals that at least some of the seeping methane was not oxidized in the zone of anaerobic oxidation of methane but entered the oxic zone. Based on the low preservation potential of lipids of endosymbiotic bacteria, such a source can be ruled out for the observed molecular fossils, but their presence reveals that oxygen-dependent consumption of methane indeed occurred at the ancient seep sites [93]. Thus, assuming that *Peregrinella* relied – in as-yet unknown ways – on chemotrophic bacteria, then its apparent preference for diffusive seepage may suggest a more prominent role of methane-oxidizing bacteria, rather than sulfide-oxidizing bacteria, in the diet of *Peregrinella*.

We infer the preference of *Peregrinella* for diffuse seepage from the larger average sizes of *Peregrinella* at sites with fewer seep cements. This may have implications for other seep-inhabiting dimerelloids from different geologic ages. In contrast to *Peregrinella*, certain dimerelloid genera including the Devonian *Dzieduszyckia* [26] and the Jurassic *Sulcirostra* [27], occur at sites where the proportion of seep cements of the limestone exceeds that seen in any *Peregrinella* site by a great margin, and may even constitute the majority of the limestone. These brachiopods potentially lived at seep sites with strong, advective fluid flow. The early Jurassic dimerelloid *Anarhynchia* cf. *gabbi* is common at a hydrothermal vent deposit [94] where sulfide most likely was the dominant reduced chemical compound. Therefore, it seems possible that the different dimerelloids developed various strategies for living at vent and seep environments. However, most of these genera have been documented from one or two seep deposits only, making a direct comparison with the trend seen in *Peregrinella* impossible.

## Conclusions

All *in situ* occurrences of *Peregrinella* investigated here were confirmed as ancient seep deposits. This supports the view that *Peregrinella* lived exclusively at seeps [19] and calls for further investigations to determine whether other dimerelloid genera that have been reported from seep or hydrothermal-vent deposits (i.e., *Anarhynchia*, *Cooperrhynchia*, *Dzieduszyckia*, *Halorella*, *Ibergirhynchia*, *Sulcirostra*) were also restricted to this type of habitat, or had broader ecologic plasticity [26].

While the question whether *Peregrinella* was chemosymbiotic or not remains unresolved, the comparative approach used here provides new insights into the paleoecology of this enigmatic brachiopod, notably the apparent preference for living at diffusive rather than advective seepage sites. Expanding this line of work to other seep-inhabiting dimerelloids could show whether this preference is shared by dimerelloids in general or whether different dimerelloid genera had adapted to vents and seeps in different ways.

Compared to other seep-inhabiting groups, especially the well-studied mollusks, *Peregrinella* has two remarkable traits: first, its quite short geologic range of only 9 m.y., and second, its extremely gregarious mode of occurrence. However, from a rhynchonellid brachiopod's perspective, these traits may not be unusual: *Peregrinella*'s stratigraphic range is not very different from the average rhynchonellid's range, and given a suitable habitat, rhynchonellid brachiopods tend to be gregarious [95]–[97]. Thus while seep mollusks clearly differ from other marine mollusks in their geologic longevity, this seems not to be the case for *Peregrinella* and perhaps also for other seep-inhabiting dimerelloids back to the Devonian.
